# Ventilator-induced lung injury: historical perspectives and clinical implications

**DOI:** 10.1186/2110-5820-1-28

**Published:** 2011-07-23

**Authors:** Nicolas de Prost, Jean-Damien Ricard, Georges Saumon, Didier Dreyfuss

**Affiliations:** 1 Assistance Publique - Hôpitaux de Paris, Hôpital Henri Mondor, Service de Réanimation Médicale, 51, Avenue de Tassigny, 94010, Créteil, France; 2 Université Paris-Diderot and PRES Sorbonne Paris Cité, Site Xavier Bichat, 75018 Paris, France; 3 Assistance Publique - Hôpitaux de Paris, Hôpital Louis Mourier, Service de Réanimation Médicale, F-92700, 178, rue des Renouillers - 92701 Colombes Cedex, France; 4 INSERM U722, F-75018 Paris, France

## Abstract

Mechanical ventilation can produce lung physiological and morphological alterations termed ventilator-induced lung injury (VILI). Early experimental studies demonstrated that the main determinant of VILI is lung end-inspiratory volume. The clinical relevance of these experimental findings received resounding confirmation with the results of the acute respiratory distress syndrome (ARDS) Network study, which showed a 22% reduction in mortality in patients with the acute respiratory distress syndrome through a simple reduction in tidal volume. In contrast, the clinical relevance of low lung volume injury remains debated and the application of high positive end-expiratory pressure levels can contribute to lung overdistension and thus be deleterious. The significance of inflammatory alterations observed during VILI is debated and has not translated into clinical application. This review examines seminal experimental studies that led to our current understanding of VILI and contributed to the current recommendations in the respiratory support of ARDS patients.

## Introduction

The prognosis of the acute respiratory distress syndrome (ARDS) has improved dramatically within the past decades, with in-hospital mortality rates ranging from 90% in the seventies [1] to approximately 30% in a recent study [2]. Reduction of the tidal volume delivered to mechanically ventilated patients, and thus of the stress applied to their lungs, unambiguously contributed to improving outcomes, as demonstrated by the ARDSnet study, which showed a 22% higher survival in patients who received lower (6 mL/kg) than in those who received larger (12 mL/kg) tidal volumes [3]. Interestingly, almost one decade before the ARDSnet study was published, the concept of "permissive hypercapnia" [4] had already led to the use of lower tidal volumes by clinicians and well-conducted observational studies had evidenced significant decrease in the mortality of patients suffering from ARDS [5]. Indeed, compelling physiological evidence had been drawn from experimental studies that had described the deleterious effects of mechanical ventilation using high peak inspiratory pressures on lungs, regrouped under the term ventilator-induced lung injury (VILI) [6-8]. In addition to this "volutrauma," so-called "low-volume" injury associated with the repeated recruitment and derecruitment of distal lung units has been incriminated in the development of VILI and forms the rationale for the use of positive end-expiratory pressure (PEEP) [9-11]. We reviewed seminal experimental studies that led to our current understanding of VILI and contributed to the current recommendations in the respiratory support of ARDS patients.

## Historical perspectives

Only 3 years after the first description of ARDS was made [12], Mead et al. developed the conceptual basis for VILI from the analysis of the mechanical properties of the lungs using a theoretical model of lung elasticity [13]. They suggested that the forces acting on lung parenchyma can be actually much greater than those applied to the airway, and theorized that the pressure tending to expand an atelectatic region at a transpulmonary pressure of 30 cm H_2_O surrounded by fully expanded lung would be approximately 140 cm H_2_O [13]. In a visionary statement, the authors concluded that "mechanical ventilation, by applying high transpulmonary pressures to heterogeneously expanded lungs, could contribute to the development of lung hemorrhage and hyaline membranes." In 1974, Webb and Tierney demonstrated for the first time that mechanical ventilation could generate lung lesions in intact animals [6]. Rats ventilated with peak inspiratory pressures of 30 or 45 cm H_2_O developed pulmonary edema within 60 and 20 min, respectively. Interestingly enough, when a 10-cm H_2_O PEEP was applied and the level of end-inspiratory pressure kept constant, the amount of lung edema was lessened [6]. Although the authors suggested that low tidal ventilation should be used, they recently mentioned that "this article seemed to interest few clinicians or investigators for a decade or more, perhaps because a similar degree of injury in patients was not apparent" and acknowledged that "in retrospect, it seems almost irresponsible that we didn't publicize our concerns that such ventilator patterns might be harmful to humans" [14]. Since this seminal publication, our knowledge of VILI has considerably increased [15].

**Figure 1:**
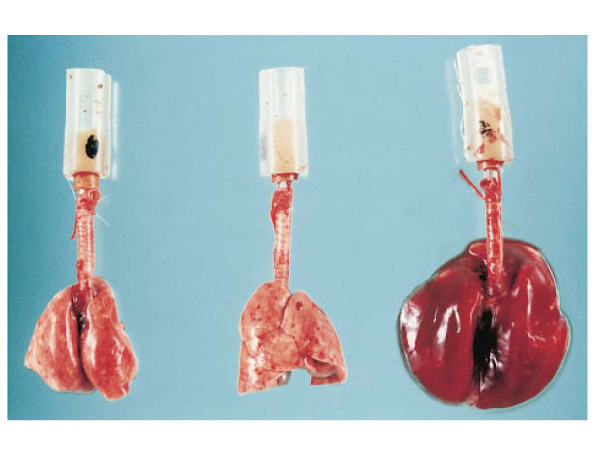
**Macroscopic aspect of rat lungs after mechanical ventilation at 45 cm H_2_O peak airway pressure**. *Left*: normal lungs; *middle*: after 5 min of high airway pressure mechanical ventilation. Note the focal zones of atelectasis (in particular at the left lung apex); *right*: after 20 min, the lungs were markedly enlarged and congestive; edema fluid fills the tracheal cannula. Used with permission. From Dreyfuss et al. [15].

**Figure 2:**
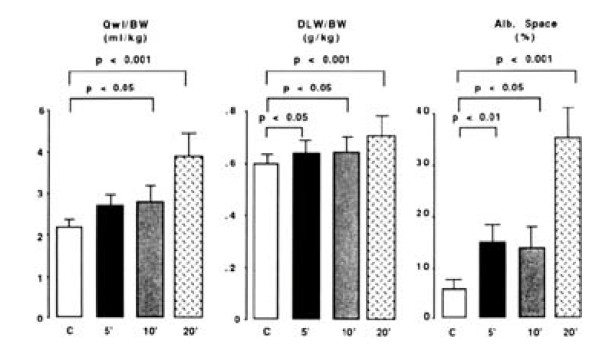
**Effect of gradual exposure of normal rats to 45 cmH_2_O peak airway pressure ventilation on lung water content and pulmonary permeability**. Pulmonary edema was assessed by measuring extravascular lung water content (Qwl/BW) and permeability alterations by measuring bloodless dry-lung weight (DLW/BW) and the distribution space in the lungs of ^125^I-labeled albumin (Alb. Space). Permeability pulmonary edema developed after only 5 minutes of mechanical ventilation. After 20 min of mechanical ventilation, there was a dramatic increase in lung water content and pulmonary permeability (*p *< 0.01 vs. other groups). Used with permission. From Dreyfuss et al. [8].

**Figure 3:**
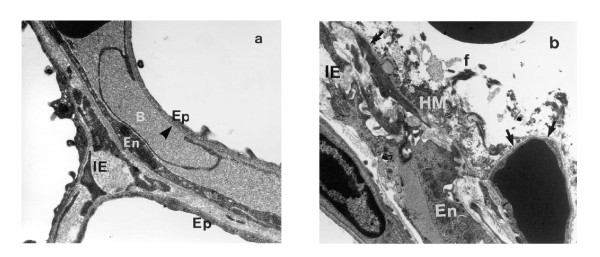
**Changes in the ultrastructural appearance of the blood-air barrier after 5 min (A) and 20 min (B) mechanical ventilation of a closed-chest rat at 45 cm H_2_O peak airway pressure**. **(A) **The thin part of an endothelial cell (En) is detached from the basement membrane (*arrowhead*) forming a bleb. **(B) **Very severe changes in the alveolar-capillary barrier resulting in diffuse alveolar damage. The epithelial layer is totally destroyed (*upper right quadrant*) leading to denudation of the basement membrane (*arrows*). Hyaline membranes (HM), composed of cell debris and fibrin (f), occupy the alveolar space. IE, interstitial edema. Used with permission. From Dreyfuss et al. [15] (panel A) and [8] (panel B).

In early publications, the development of pulmonary edema during mechanical ventilation was ascribed to increased lung microvascular pressure, resulting from high lung volume ventilation and surfactant depletion [6,7,16]. As a matter of fact, Parker et al. showed that mean lung microvascular pressure increased by 12.5 cm H_2_O during ventilation of open-chest dogs at 64 cm H_2_O positive inspiratory pressure [16]. Indeed, it has been demonstrated that lung inflation decreases interstitial pressure (thus, increases transmural pressure) around extra-alveolar vessels because of the interdependence phenomenon and around alveolar vessels because of surfactant inactivation. Inflating lungs dilates extra-alveolar vessels [17] and during inflation from low transpulmonary pressure, the increase in vessel diameter is such that an effective outward-acting pressure in excess of pleural pressure (1 to 2 cm H_2_O for each centimeter of water increase in transpulmonary pressure) expands these vessels [18]. Surfactant is inactivated commensurately with the magnitude of tidal volume and duration of ventilation during ventilation of excised lungs [19-21]. It was demonstrated that the increase in alveolar surface tension resulting from surfactant inactivation leads to increased filtration through alveolar microvessels [22-24]. However, the magnitude of microvascular pressure changes during lung inflation is modest and insufficient to explain the occurrence of severe pulmonary edema during mechanical ventilation with a high tidal volume. In addition to pressure changes, alterations of alveolo-capillary barrier permeability are involved in the development of pulmonary edema during high-volume ventilation of intact animals and are the most important responsible for VILI. The increase in alveolar epithelial permeability to small hydrophilic solutes has been studied by Egan during static inflation of fluid-filled *in situ *sheep lobes [25,26]. The equivalent-pore radius (an index of epithelial permeability) increased from approximately 1 nm at 20 cm H_2_O inflating pressure to 5 nm at 40 cm H_2_O alveolar pressure. Albumin diffused freely across the epithelium at the highest pressures, indicating the presence of large leaks. Such permeability increases persisted or even increased after cessation of inflation, implying that epithelial injury was irreversible. Unexpectedly, Parker et al. demonstrated in isolated blood-perfused dog lobes that microvascular permeability alterations, as assessed by increased capillary filtration coefficient, also occurred during high peak pressure ventilation (> 45 cm H_2_O) [7]. It was subsequently demonstrated that, within minutes after the onset of ventilation, rats ventilated with 45 cm H_2_O peak pressures exhibited not only macroscopic pulmonary edema (Figure 1) but also a dramatic increase in microvascular permeability assessed by the distribution space of intravenously injected ^125^I-labeled albumin (Figure 2) [8]. Electron microscopy studies consistently revealed widespread disruption of epithelial cells leading to denudation of basement membranes and the presence of many gaps in the capillary endothelium (Figure 3A, B) [8]. Such findings demonstrated that high-volume mechanical ventilation leads to pulmonary edema of the permeability type.

The increase in transmural microvascular pressure, even if modest, contributes to the severity of pulmonary edema, which may be fulminating during VILI, because any increase in the driving force will have a dramatic effect on edema formation in the face of an altered microvascular permeability [27].

## Mechanical determinants of ventilator-induced lung injury

### Role of end-inspiratory lung volume

**Figure 4:**
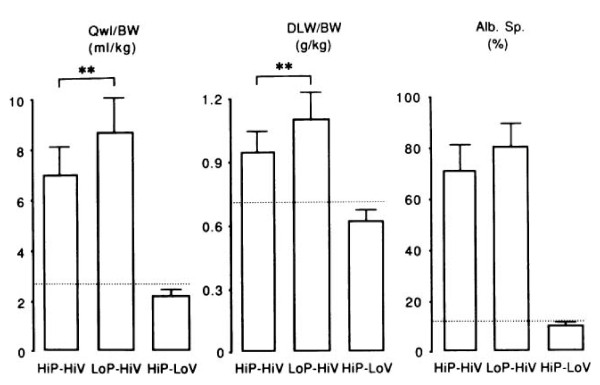
**Comparison of the effects of high peak (45 cm H_2_O) positive inspiratory pressure plus high tidal-volume ventilation (HiP-HiV) with the effects of negative inspiratory airway pressure plus high tidal-volume ventilation (iron lung ventilation = LoP-HiV) and of high peak (45 cm H_2_O) positive inspiratory pressure plus low tidal-volume ventilation (thoracoabdominal strapping = HiP-LoV)**. Pulmonary edema was assessed by the determination of extravascular lung water content (*Qwl/BW*) and permeability alterations by the determination of bloodless dry-lung weight (*DLW/BW*) and of the distribution space in the lungs of ^125^I-labeled albumin (*Alb. Sp*.). Dotted lines represent the upper 95 percent confidence limit for control values. Permeability edema occurred in both groups receiving high tidal-volume ventilation. Animals ventilated with a high peak-pressure and a normal tidal volume had no edema. Used with permission. From Dreyfuss et al. [29].

**Figure 5:**
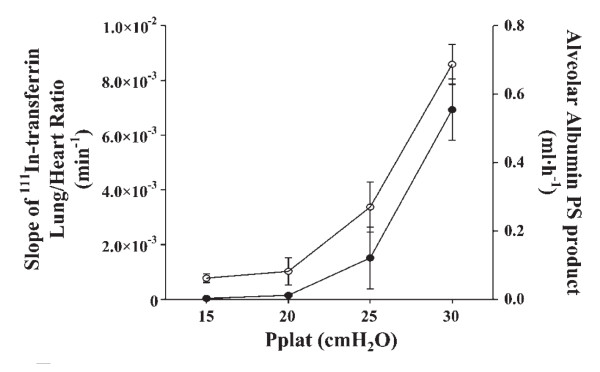
**Relationship between plateau pressure (*Pplat*) and ^111^In-transferrin lung-to-heart ratio slope (an index of lung microvascular permeability; left axis, open circles) and alveolar ^99 m^Tc-albumin permeability-surface area product (an index of alveolar epithelium permeability; right axis, full circles) in mechanically ventilated rats**. Both indexes dramatically increased for plateau pressures comprised between 20 and 25 cm H_2_O. Used with permission. From de Prost et al. [32].

The term "barotrauma" was formerly used by clinicians to describe the lung damage attributable to ventilation with high peak pressures; the most common form is pneumothorax [28]. However, it was proposed that this term should be replaced by the term "volutrauma." To discriminate between the effects of lung distension and airway pressure, rats ventilated with identical (45 cm H_2_O) peak pressures using high or low volume (generated by limiting thoracoabdominal excursions by strapping) ventilation were compared [29]. The rats subjected to high volume-high pressure ventilation developed pulmonary edema whereas those subjected to low volume-high pressure ventilation did not. That high pressures are not a prerequisite for the development of pulmonary edema was further confirmed by ventilating rats with high tidal volume but negative airway pressures by means of an iron lung (Figure 4) [29]. Those findings have been replicated in rabbits [30] and in lambs [31]. The question of whether pulmonary edema during mechanical ventilation occurs above a threshold volume was addressed by Carlton et al. in a lamb model of VILI [31]. Gradually increasing tidal volumes, corresponding to end-inspiratory pressures of 16, 33, 43, and 61 cm H_2_O, these authors showed that lung lymph flow and protein concentration were increased only when the highest tidal volume (57 mL/kg) and pressure level (61 cm H_2_O) were reached, suggesting that microvascular alterations in response to overinflation occurred beyond a volume/pressure threshold rather than gradually [31]. Those findings were confirmed using scintigraphic methods that allow for the assessment of simultaneous changes in alveolar and microvascular permeability during lung inflation in rats with previously intact lungs [32]. Interestingly, the same end-inspiratory pressure threshold (between 20 and 25 cm H_2_O, corresponding to tidal volumes of 13.7 ± 4.69 and 22.2 ± 2.12 mL/kg) was observed for epithelial and endothelial permeability changes (Figure 5).

**Figure 6:**
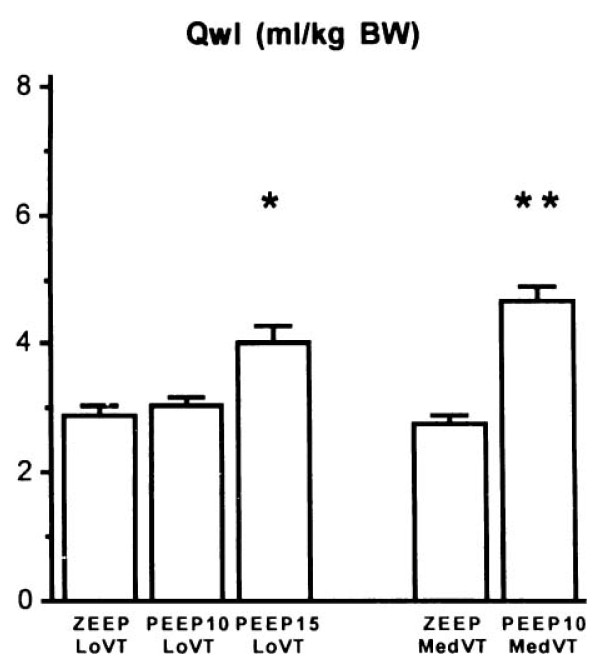
**Effect of increasing PEEP from 0 to 15 cm H_2_O during ventilation with two levels of tidal volume (V_T_, 7 ml/kg of body weight = Lo V_T_; 14 ml/kg BW of body weight = Med V_T_)**. When PEEP was increased, pulmonary edema, as evaluated by extravascular lung water (*Qwl*), occurred. The level of PEEP required to produce edema varied with V_T_; 15 cm H_2_O PEEP during ventilation with a low V_T _versus 10 cm H_2_O PEEP during ventilation with a moderately increased V_T_. *p < 0.05; **p < 0.01 vs. ZEEP and the same V_T_. Used with permission. From Dreyfuss and Saumon [33].

Increasing end-inspiratory volume by increasing the functional residual capacity (i.e., applying PEEP) may cause lung injury independently of tidal volume [33]. As a result, PEEP application when tidal volume is kept constant increases lung end-inspiratory volume and can be deleterious. For instance, rats ventilated with a tidal volume within the physiologic range developed pulmonary edema when a 15 cm H_2_O but not a 10 cm H_2_O PEEP was applied [33]. Likewise, there was no effect of doubling tidal volume in animals ventilated with ZEEP, but it resulted in pulmonary edema when a 10 cm H_2_O PEEP was used (Figure 6) [33].

### Low lung-volume injury and beneficial effects of PEEP

The application of PEEP results in less severe lung lesions when end-inspiratory volume is kept constant. This might be related to a reduction of tidal volume and the stabilization of terminal units. Webb and Tierney showed that at 45 cm H_2_O teleinspiratory pressure, edema was less severe when a 10 cm H_2_O PEEP was applied and attributed this effect to the preservation of surfactant activity [6]. It was later confirmed that for a same end-inspiratory pressure, rats ventilated with zero end-expiratory pressure exhibited larger amounts of lung edema, as determined by extravascular lung water measurement, than those ventilated with PEEP. However, in the presence of PEEP edema remained confined to the interstitium, whereas there was alveolar flooding in its absence [29]. The application of PEEP during VILI development was associated with a preservation of the integrity of the alveolar epithelium and the only ultrastructural abnormalities observed were endothelial blebbing and interstitial edema [29]. This beneficial effect of PEEP might be related to the reduction of cyclic recruitment-derecruitment of lung units, which causes the abrasion of the epithelial airspace lining by interfacial forces [10,11]. These phenomena of repeated opening and closing of distal lung units have been theorized to provide an explanation why large cyclic changes in lung volume promote the development of edema. Indeed, for an identical increase in mean airway pressure, ventilation of hydrochloric acid-injured dog lungs with a large tidal volume and a low PEEP resulted in more severe edema than did ventilation with a small tidal volume and a high PEEP [34]. The effect of the amplitude of tidal volume on alveolar epithelium protein permeability, end-inspiratory pressures being kept constant by manipulating PEEP level, was further confirmed in rats using noninvasive scintigraphic techniques [35]. The alveolar albumin permeability-surface area product, measured from the clearance of an intra-tracheally instilled ^99 m^Tc-labeled albumin solution, dramatically increased, and in a dose-dependent manner, when V_T _was increased from 8 to 24 and 29 mL/kg [35]. Finally, the decrease in cardiac output secondary to the increase in intrathoracic pressures has been demonstrated to account for a part of the PEEP-induced reduction in pulmonary edema [33]. All in all, the beneficial effects of PEEP during high-volume ventilation (i.e., reductions in both the amount of edema and the severity of cell damage) involve a combination of hemodynamic alterations, shear stress reduction, and surfactant modifications.

### Influence of previous injury on the susceptibility to VILI

**Figure 7:**
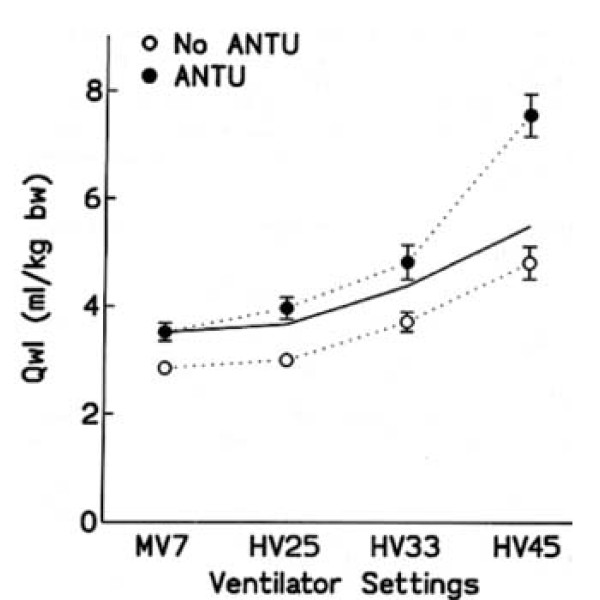
**Interaction between previous lung alterations and mechanical ventilation on pulmonary edema**. Effect of previous toxic lung injury. Extravascular lung water (*Qwl*) after mechanical ventilation in normal rats (*open circles*) and in rats with mild lung injury produced by *α*-naphthylthiourea (ANTU) (*closed circles*). Tidal volume (V_T_) varied from 7 to 45 ml/kg body weight. The solid line represents the Qwl value expected for the aggravating effect of ANTU on edema caused by ventilation, assuming additivity. ANTU did not potentiate the effect of ventilation with V_T _up to 33 ml/kg body weight. In contrast, V_T _45 ml/kg body weight produced an increase in edema that greatly exceeded additivity, indicating synergy between the two insults. Used with permission. From Dreyfuss et al. [38].

This is an important aspect of VILI. In fact, original descriptions of VILI were made on normal lungs subjected to very high distending pressure [6-8,36]. It is fundamental to assess whether a preexisting injury may sensitize lungs to the deleterious effects of mechanical ventilation. This possibility was suggested by the calculations made by Mead and colleagues [13], who showed that the pressure tending to expand an atelectatic region surrounded by a fully expanded lung is approximately 140 cm H_2_O at a transpulmonary pressure of 30 cm H_2_O. Several studies evaluated the susceptibility to VILI of previously injured lungs. For instance Parker's group showed that neither low doses of oleic acid nor 25 cm H_2_O peak inspiratory pressure mechanical ventilation increased filtration coefficient and wet-to-dry ratio in an isolated-perfused rabbit lung model [37]. However, the combination of both injuries did so and was thus more deleterious than either one alone. These observations were later confirmed and expanded. The effects of different degrees of lung distention were studied in rats whose lungs had been injured by α-naphthylthiourea (ANTU) [38]. Low doses of ANTU were used to create mild lung injury. As a matter of fact, ANTU infusion alone caused moderate interstitial pulmonary edema of the permeability type. When used in the absence of ANTU administration, mechanical ventilation resulted in a permeability edema, which was more severe as tidal volume was increased. The combination of both injuries showed that they were not simply additive but synergistic. Indeed, the severity of edema was more important that the simple summation of the effect of either one alone (Figure 7). Interestingly, alterations of lung mechanical properties induced by ANTU administration were predictive of this synergism [38]. This finding underlines the importance of an adequate examination of the pressure-volume curve during VILI.

### Interest of the pressure-volume curve

#### Lung compliance and upper inflection point

Because of the heterogeneity of lung volume reduction, ventilation will be redistributed toward the more compliant zones, which may favor their overinflation. In the absence of an accurate tool for measuring ventilatable lung volume, analysis of the pressure-volume (PV) curve may help understand how pre-existing lung injury interacts with ventilator-induced injury. The decrease in compliance associated with lung edema is related to the reduction in the ventilatable lung volume, the so-called "baby lung" observed during ARDS [39,40]. In addition, the upper inflection point (UIP) of the PV curve represents the lung volume at which lung compliance begins to diminish and thus is a surrogate of the beginning of overinflation [41,42]. Both respiratory system compliance and the position of the UIP may allow for a better understanding of the consequences of high-volume ventilation. Indeed, the amount of pulmonary edema produced by high-volume ventilation in animals given ANTU [38] was inversely proportional to the respiratory system compliance measured during the first breaths, i.e., before any damage due to ventilation had occurred. In other words, the lower the lung compliance after ANTU infusion, the higher the amount of edema during VILI. Similarly, animals with an UIP occurring at lower pressures (indicating an earlier onset of overdistension) developed more edema than those with an UIP occurring at higher pressures. This suggests that reduced lung distensibility predisposes to the noxious effects of high volume ventilation.

This concept was further strengthened during experimental reduction of the ventilatable lung volume by instillation of a viscous liquid in distal airways of rats. As with ANTU, the higher the compliance and volume of the UIP after instillation of the liquid but before the onset of high-volume ventilation, the lower was the amount of edema observed after high peak pressure ventilation, suggesting that the UIP is a marker of the amount of ventilatable lung volume, and a predictor of the development of edema during mechanical ventilation [43].

#### Lower inflection point (LIP): can lung parenchyma be altered by repetitive opening and closure of distal airway units?

The lower inflection point (LIP) of the PV curve corresponds to the volume and pressure at which there is the greatest increase in the compliance of the respiratory system. This point may reflect the reexpansion of atelectatic parenchyma and has been considered to indicate the minimal pressure required to recruit collapsed alveoli, as setting the PEEP level according to this point has been shown to improve oxygenation of ARDS patients [44,45]. Sykes' group showed that setting PEEP above the LIP in rabbit models of surfactant depletion (after repeated alveolar lavage) improved oxygenation and lessened lung damage compared with lower PEEP levels [9,46]. This lessening of pathological alterations was observed even with ventilator settings that achieved identical mean airway pressures in the low and high PEEP groups [46]. These observations were confirmed in isolated surfactant-depleted nonperfused lungs [11]. In contrast, those findings could not be replicated by Sykes' group in a rabbit model of hydrochloric acid instillation [47], suggesting that this strategy for setting PEEP based on the LIP of the pressure-volume curve is only beneficial in conditions associated with major alveolar instabilities, such as encountered during surfactant depletion. Moreover, Lichtwarck-Aschoff et al. showed that when PEEP was set at the LIP in surfactant-depleted piglets, there was a decrease in compliance during tidal volume insufflation, which indicated overinflation [48]. The authors concluded that the PEEP level that allows compliance to remain constant during the full tidal volume insufflation cannot be routinely derived from analysis of the pressure-volume curve.

These discrepancies are not trivial because they underlie the concept of "protective ventilation" during acute lung injury. Indeed, whereas numerous experimental studies have demonstrated that lung overinflation--regional or global--leads to VILI [6-8,36,49], the genesis of lesions at low lung volume is much more debated [50]. Such lesions could result from repetitive opening and collapse of distal airways/alveoli, a mechanism termed "atelectrauma" [51]. However, as explained earlier, this phenomenon might be limited to certain particular settings (e.g., surfactant depletion) and might not be relevant to edematous lungs. For instance, Hubmayr's group challenged this concept using both elegant experimental settings and insightful mathematical models [50,52]. They concluded that distal airways do not close and open during ventilation when PEEP is set below the LIP but, instead, demonstrated that the LIP reflects the movement of liquid or foam in the airways: when a liquid column is present in the airways, it opposes a marked resistance to the airflow; after a certain pressure threshold the liquid is propelled into the alveoli where it can distribute in a much larger volume than in the airways. As a result, there is an abrupt gain in volume at constant (or even decreased) pressure that translates into a prominent knee on the PV curve. In such circumstances, no epithelial lesion is generated and the LIP may be considered as an artifact. There is no doubt that a certain level of PEEP is beneficial during VILI [6,9,29,46], but there is no firm demonstration that this level must necessarily be "high" rather than "low" and may be deduced from the presence of a LIP on the PV curve. Interestingly, this controversy about the respective contribution of overall lung distension and of cyclic recruitment-derecruitment has its exact counterpart for the management of ARDS: it is beyond doubt that tidal volume reduction saves lives [3], whereas the improvement of prognosis with higher PEEP is highly disputable [2,53,54]. This clinical controversy will be addressed elsewhere in this article.

## The biotrauma hypothesis

**Figure 8:**
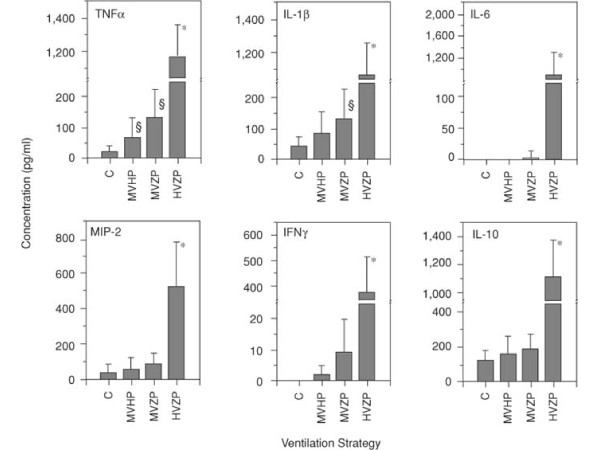
**Effect of different ventilator strategies on cytokine concentrations in lung lavage of isolated unperfused rat lungs**. Four ventilator settings were used: controls (*C *= normal tidal volume), moderate tidal volume + high PEEP (*MVHP*), moderate tidal volume + zero PEEP (*MVZP*), high tidal volume + zero PEEP (*HVZP*) resulting in the same end-inspiratory distension as MVHP. Major increases in cytokine concentrations were observed with HVZP. Used with permission. From Tremblay et al. [57].

**Figure 9:**
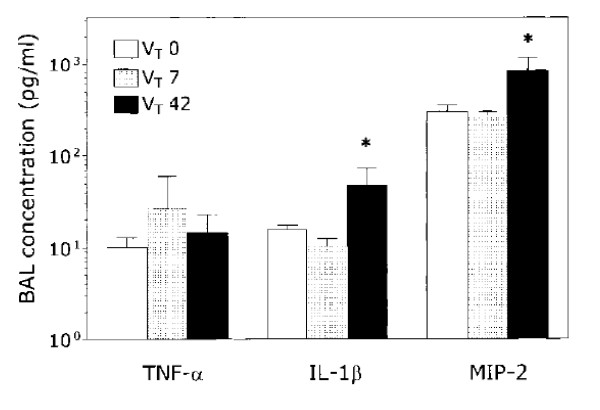
**TNF-α, IL-1β, and MIP-2 concentrations in bronchoalveolar lavage fluid of isolated, nonperfused rat lungs maintained for 2 h in a statically inflated state at 7 cm H_2_O airway pressure (*V_T_0*), ventilated with 7 mL/kg tidal volume and 3 cm H_2_O positive end-expiratory pressure (*V_T_7*), or ventilated with 42 mL/kg V_T _and zero end-expiratory pressure (*V_T_42*)**. IL-1β and MIP-2 concentrations were slightly higher (*p < 0.05) in the V*T*42 group. There was no difference in TNF-α concentration. Used with permission. From Ricard et al. [59].

**Figure 10:**
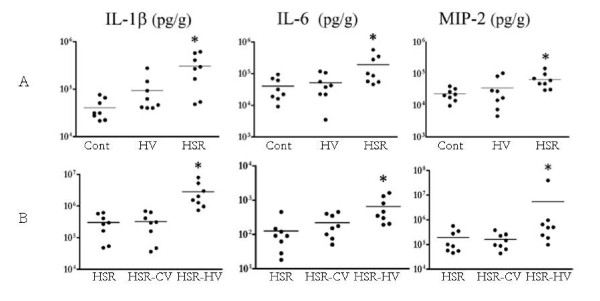
**(A) Comparison of lung cytokine levels in nonventilated rats (*Cont*) and in rats subjected to an injurious mechanical ventilation strategy (30 mL/kg tidal volume and zero end-expiratory pressure) alone (*HV*) or to hemorrhagic shock-reperfusion alone (*HSR*)**. Compared with controls, lung cytokine concentrations were higher after HSR but not after HV. **(B) **Comparison of lung cytokine levels in rats subjected to HSR alone, HSR combined with conventional ventilation (*HSR-CV*) and HSR followed by HV (*HSR-HV*). Injurious ventilation (*HV*) after HSR significantly increased mediator release above the levels observed after HSR alone or combined with conventional ventilation. Results are expressed in pg/g. *p < 0.05 as compared with controls **(A) **or HSR **(B)**. The same observations were made in bronchoalveolar lavage fluid and in plasma. Adapted with permission. From Bouadma et al. [60].

Several studies have shown that protracted mechanical ventilation using high peak pressures led to lung infiltration with neutrophils [49,55]. Moreover, neutrophil depletion was associated with better gas exchange and less lung injury in a rabbit model of surfactant depletion [56], suggesting that the inflammatory reaction per se could be deleterious. On the other hand, early studies had evidenced that mechanical ventilation could have deleterious systemic effects. For instance, Kolobow et al. demonstrated that sheep ventilated with 50 cm H_2_O peak pressure, corresponding to tidal volumes of 50 to 70 mL/kg, died from multiple organ dysfunction within 48 h [36]. The biotrauma hypothesis, i.e., lung tissue stretching might result in lung damage solely through the release of inflammatory mediators and leukocyte recruitment, has been put forward to provide an explanation why most patients with ARDS die from multiple organ failure rather than hypoxemia. Tremblay et al. [57] showed in unperfused rat lungs that high tidal volume ventilation (40 mL/kg) with zero end-expiratory pressure resulted in dramatic increases in the lung lavage levels of tumor necrosis factor-α (TNF-α), interleukin-1β (IL-1β), interleukin-6, and macrophage inflammatory protein-2, compared with controls (Figure 8), suggesting that mechanical ventilation can influence the inflammatory/anti-inflammatory balance in the lungs. However, using the same model of unperfused rat lungs, others found only slightly higher IL-1β and MIP-2 bronchoalveolar lavage fluid concentration in rats ventilated with 42 mL/kg tidal volume than in those ventilated with 7 mL/kg tidal volume [58,59] (Figure 9). Moreover, there was no difference in TNF-α lung level between both ventilator strategies. The authors concluded that ventilator strategies that injure lungs do not necessarily result in primary production of proinflammatory cytokines in the lungs [59]. The two-hit hypothesis has been put forward to reconcile these discrepant findings. Injurious mechanical ventilation may not be sufficient *per se *to promote intense lung proinflammatory cytokine secretion but will do so in combination with another aggression [60,61]. For instance, hemorrhagic shock and resuscitation and high PIP ventilation in rats interact to increase lung and systemic release of proinflammatory mediators (Figure 10). The biotrauma hypothesis seemed supported by the finding that ARDS patients ventilated with a protective strategy (i.e., tidal volume of 7 mL/kg and PEEP of 15 cm H_2_O determined from analysis of the PV curve) exhibited lower bronchoalveolar lavage fluid and plasma concentrations of inflammatory mediators than patients ventilated with a "control" strategy (i.e., tidal volume of 11 mL/kg and PEEP of 6 cmH_2_O) only 36 h after randomization [62]. However, this study was not designed to address whether these decreases were associated with improved clinical endpoints and the clinical meaning of these changes in cytokine levels remains elusive. Similarly, modest, although significant, differences were observed in the plasma levels of IL-6 and IL-8 in ARDS patients ventilated with 6 vs. 12 mL/kg tidal volume [63]. In contrast, transiently changing ventilatory settings from a low tidal volume (5 mL/kg)-high PEEP (15 cm H_2_O) to a high tidal volume (12 mL/kg)-low PEEP (5 cm H_2_O) strategy in ALI patients led to an increase in plasma and bronchoalveolar lavage fluid levels of cytokines [64]. Intriguingly, most of these were anti-inflammatory cytokines (IL-1 receptor antagonist and IL-10), emphasizing that it remains unclear whether mechanical ventilation affects the balance of cytokines toward pro- or anti-inflammation [65].

**Figure 11:**
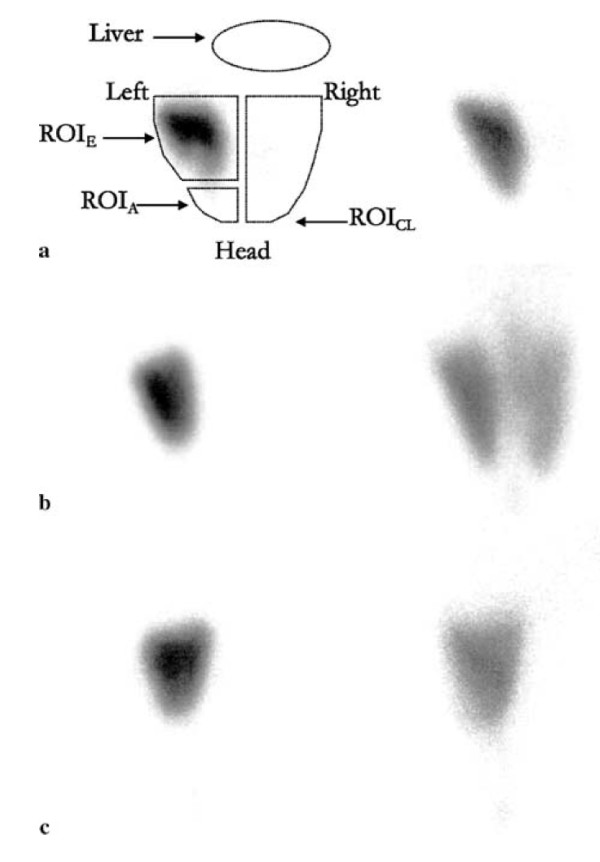
**Examples of scintigraphy images integrating the 15 min following tracer instillation (t0-t15; *left panels*) and the last 15 min of the experiment (t195-t210; *right panels*)**. Regions of interest (ROIs) were drawn around initial focus of edema (ROI_E_), the apex of the same lung (ROI_A_), the contralateral lung (ROI_CL_), and over the thorax (ROI_T_). At baseline (*left panels*), all animals were ventilated with a tidal volume of 8 mL/kg and a PEEP of 2 cmH_2_O and exhibited focalized localization of the tracer in the left lung. When the same ventilator settings were kept during the experiment **(a)**, the tracer remained remarkably confined to the initial zone; there was no contralateral and slight homolateral dissemination. High-volume ventilation (Pplat = 30 cmH_2_O) with no PEEP **(b) **induced strong homo- and contralateral dispersion of the tracer and systemic leakage, as attested by the evident decrease in overall activity. High-volume ventilation with 6 cmH_2_O PEEP **(c) **induced systemic, but not contralateral, dissemination of the tracer. Used with permission. From de Prost et al. [35].

Finally, there is growing evidence that ventilator settings may localize or disperse proteinaceous lung edema or bacteria [66-68]. Indeed, ventilation with high tidal volume and no PEEP promoted contralateral bacterial seeding in a unilateral model of *Pseudomonas aeruginosa *pneumonia in rats [68]. In contrast, ventilation at the same end-inspiratory pressure but with a high PEEP and thus a lower tidal volume prevented such contralateral dissemination. The potential for adverse ventilator patterns to disperse localized alveolar edema to the opposite lung was studied using scintigraphic methods [35]. A ^99 m^Tc-labeled albumin solution was instilled in a distal airway and produced a zone of alveolar flooding that stayed localized during conventional ventilation. Ventilation with high end-inspiratory pressure dispersed alveolar liquid in the lungs. This dispersion began almost immediately after high-volume ventilation was started and was likely the consequence of a convective movement induced by ventilation. Interestingly, PEEP application prevented this spread even when tidal volume was equivalent and thus end-inspiratory pressure higher (Figure 11). In heterogeneously ventilated lungs, fluid transfer may be propelled toward regions of normal compliance. PEEP may prevent fluid dispersion by avoiding lung collapse and stabilizing edema in the distal airways.

## Modulation of VILI

Reducing the stress applied to the lungs by lowering tidal volume improved the survival of ARDS patients [3]. However, the mortality rate remains high, between 30 and 60% depending on the study [2,69]. Thus, a considerable amount of studies aiming at developing new ventilator strategies or pharmacological treatments of VILI have been published.

### Mechanical measures

Despite promising experimental results that suggest that they could suppress air-liquid interfaces and allow for reopening of collapsed or liquid-filled areas, surfactant administration [70] and partial liquid ventilation with perfluorocarbons [71] have been abandoned since the negative results of clinical trials. Synthetic surfactant administration failed to improve oxygenation [72] and to improve lung mechanics [73] in ARDS patients. This could be related to the type of surfactant tested as another study using a natural surfactant in a pediatric population with acute lung injury was associated with increased survival [74]. Partial liquid ventilation with perfluorocarbons at both "high" (20 mL/kg) and "low" (10 mL/kg) doses did not improve outcome of ARDS patients [75]. Such negative results might have been anticipated from the results of experimental studies that had previously demonstrated that ventilator-induced pulmonary edema was aggravated in animals given such high doses of perfluorocarbons because they favored gas trapping in the distal lung [76].

Two randomized, controlled trials showed no effect of prone positioning on outcome of ARDS patients [77,78]. However, Mancebo et al. demonstrated that prone positioning was associated with a trend toward higher survival when administered early during the course of the disease and for as much as 20 h per day [79]. A recent meta-analysis found that prone positioning was associated with improved mortality in the most hypoxemic patients (i.e., having a PaO_2_/FiO_2 _ratio < 100 mmHg) [80]. These results are in keeping with experimental studies that evidenced that prone ventilation lessened the histological injury associated with high peak pressure ventilation in a dog model of oleic-acid lung injury [81]. These protective effects likely stem from a more homogenous distribution of ventilation associated with prone ventilation [82].

### Pharmacological treatments

Numerous cell signaling pathways are involved in the pathophysiology of VILI. As such, hundreds of studies aiming at testing pharmacologic interventions during VILI have been published: 1) studies designed to modulate microvascular permeability using blockers of stretch-activated cation channels [83], beta-adrenergic agonists [84], inhibitors of phosphotyrosine kinase [85], or reducing myosin light chain phosphorylation with adrenomedullin [86]; 2) studies testing the modulation of the imbalance between pro- and anti-inflammatory mediators in the lung. For instance, the administration of anti-TNF-α antibody [87-89] and the inhibition of MIP-2 activity [90,91] reduced neutrophilic infiltration and lung injury; and 3) studies modulating hormonal and metabolic pathways: inhibition of the renin-angiotensin system [92,93] and pretreatment with atorvastatin or simvastatin [94,95] decreased alveolar-capillary barrier permeability and lung inflammation in experimental models of VILI. However, none of those pharmacological interventions has proven beneficial for the prevention and treatment of VILI in patients. Although it is probably illusory to believe that a single pharmacological intervention might be beneficial in patients, the description of those pathways illustrates the complexity of the cellular mechanisms involved in VILI.

## Clinical relevance of VILI

As discussed earlier in this article, the prevention of ventilator-associated lung injury during treatment of ALI may theoretically stem from two approaches: 1) easing the strain [96] applied to diseased lungs through the reduction of tidal volume and therefore of end-inspiratory lung volume (which may be evaluated indirectly by inspiratory plateau pressure); and 2) reducing the so-called atelectrauma via an adequate use of PEEP. Whereas the first concept, which is undisputed on physiological grounds, received a resounding illustration with the demonstration of an improved prognosis by a simple reduction of tidal volume in ARDS patients [3], things are far less clear regarding the second. Indeed, as explained above, the concept of repetitive opening and closure or distal airspaces and the putative low lung volume lesions related to insufficient PEEP levels remains debated. This uncertainty formed the basis of three high-quality, independent, randomized, controlled studies [2,53,54], which all failed to demonstrate improved survival with a high PEEP strategy. Although a meta-analysis of these trials showed a reduction of mortality using higher PEEP levels in the most severe population [97], this does not constitute a definite proof but rather a hypothesis that would require another well-conducted, randomized trial to be confirmed. Moreover, the marked heterogeneity of ventilator protocols among these three studies (with resulting considerable differences in plateau pressures between one study [54] and the others [2,53]) casts some doubt on the physiological rationale for this meta-analysis. Interestingly, a recent study using positron emission tomography imaging in ARDS patients to quantify lung metabolism, a surrogate of lung inflammation, showed that lung regions undergoing cyclic recruitment-derecruitment did not exhibit higher metabolism than those continuously collapsed throughout the respiratory cycle, thus questioning the concept of low lung volume injury [98].

## Conclusions

Few experimental concepts have led to dramatic changes in clinical practices as the concept of VILI did. The understanding that end-inspiratory distension is the main determinant of VILI led to the reduction of tidal volume and improved the survival of ARDS patients. Yet, whether tidal volume should be set at 6 mL/kg in all patients remains unsettled [99], and there is currently no bedside tool that allows an accurate assessment of the aerated lung volume and thus no way to tailor tidal volume routinely. Rather than sticking to arbitrarily fixed approaches, clinicians should tailor mechanical ventilation according to patient's individual characteristics, including close monitoring of plateau pressure and maybe PEEP titration guided by esophageal pressure monitoring [100]. In addition, opinion leaders should strive to convince clinicians not to use excessive tidal volume, which is still unfortunately the case [101]. Indeed, two epidemiological studies suggested an association between ventilator settings (i.e., use of a tidal volume > 6 mL/kg or 700 mL [102] and plateau pressure > 30 cm H_2_O [103]) and the development of ARDS in mechanically ventilated patients who did not meet ARDS criteria upon hospital admission. Similarly, a relationship between the use of large tidal volumes and postoperative respiratory failure has been established after mechanical ventilation in patients undergoing a pneumonectomy, suggesting that ventilation does not need to be protracted to be deleterious [104]. Even though the tidal volume to be used in patients with so-called normal lungs has not been defined yet, these studies suggest that protective ventilator settings could prevent the development or ARDS, particularly when a systemic insult is associated.

The story of VILI and its clinical correlates may be viewed as a success of applied physiology [105,106]. Indeed, based on sound physiology, physicians had started to reduce tidal volume long before this strategy was proved by randomized, controlled trials [5,107]. The considerable improvement in survival of ARDS patients with a simple and low-cost physiologic approach is at striking contrast with the failure of many randomized trials of expensive drugs to improve the prognosis of critically ill patients [105,106]. Nevertheless, many questions persist and VILI will likely not soon be out of the spotlight.

## Abbreviations

ANTU: α-naphthylthiourea; ARDS: acute respiratory distress syndrome; LIP: lower inflection point; PEEP: positive end-expiratory pressure; PV: pressure-volume; UIP: upper inflection point; VILI: ventilator-induced lung injury.

## Competing interests

The authors declare that they have no competing interests.

## Authors' contributions

NDP and DD drafted the manuscript, and JDR and GS revised the manuscript.
